# Enhancing the biosynthesis of polyunsaturated fatty acids by *Rhodotorula mucilaginosa* and *Lodderomyces elongisporus*

**DOI:** 10.1186/s40643-024-00755-7

**Published:** 2024-04-18

**Authors:** Amera A. Abaza, Yousseria M. Shetaia, Noha M. Sorour, Ashraf S. A. El-Sayed, Ashraf F. El-Baz

**Affiliations:** 1 Department of Industrial Biotechnology, Genetic Engineering and Biotechnology Research Institute (GEBRI), University of Sadat City, Sadat City, 22857/79 Egypt; 2 Department of Microbiology, Faculty of Science, Ain Shams University, Cairo, 11566 Egypt; 3 Enzymology and Fungal Biotechnology Lab (EFBL), Botany and Microbiology Department, Faculty of Science, Zagazig University, Zagazig, 44519 Egypt

**Keywords:** Eicosapentaenoic acid (EPA), Eicosadienoic acid (EDA), *Lodderomyces elongisporus*, *Rhodotorula mucilaginosa*, Two-stage culture

## Abstract

Single-cell oils (SCO) produced by oleaginous yeast hold promise as a sustainable alternative for producing nutritionally and pharmaceutically valuable lipids. However, the accumulation of oils varies substantially between yeast spp. Consequently, identifying well-suited producers with a high innate capacity for lipids biosynthesis is paramount. Equally important is optimizing culturing and processing conditions to realize the total lipids production potential of selected strains. The marine *Rhodotorula mucilaginosa* and *Lodderomyces elongisporus* yeast were investigated to explore their potential for polyunsaturated fatty acids (PUFAs) production on high glucose media (HGM) using two-stage culture mode. Both strains accumulated > 20% (w/w) of their dry cell weight as lipids when grown on HGM using a two-stage culture system. Both yeast isolates exhibited a maximal lipid/biomass coefficient (Y_L/X_) of 0.58–0.66 mg/mg at 7 °C and 0.49–0.53 mg/mg at 26 °C when grown on 8% glucose and produced monounsaturated and PUFAs similar to that of Menhaden and Salmon marine oils. For the first time, significant amounts of Eicosapentaenoic acid (19%) and Eicosadienoic acid (19.6%) were produced by *L. elongisporus* and *R. mucilaginosa*, respectively. Thus, the SCO derived from these wild strains possesses significant potential as a substitute source for the industrial-scale production of long-chain PUFAs, making them a promising contender in the market.

**Graphical Abstract:**

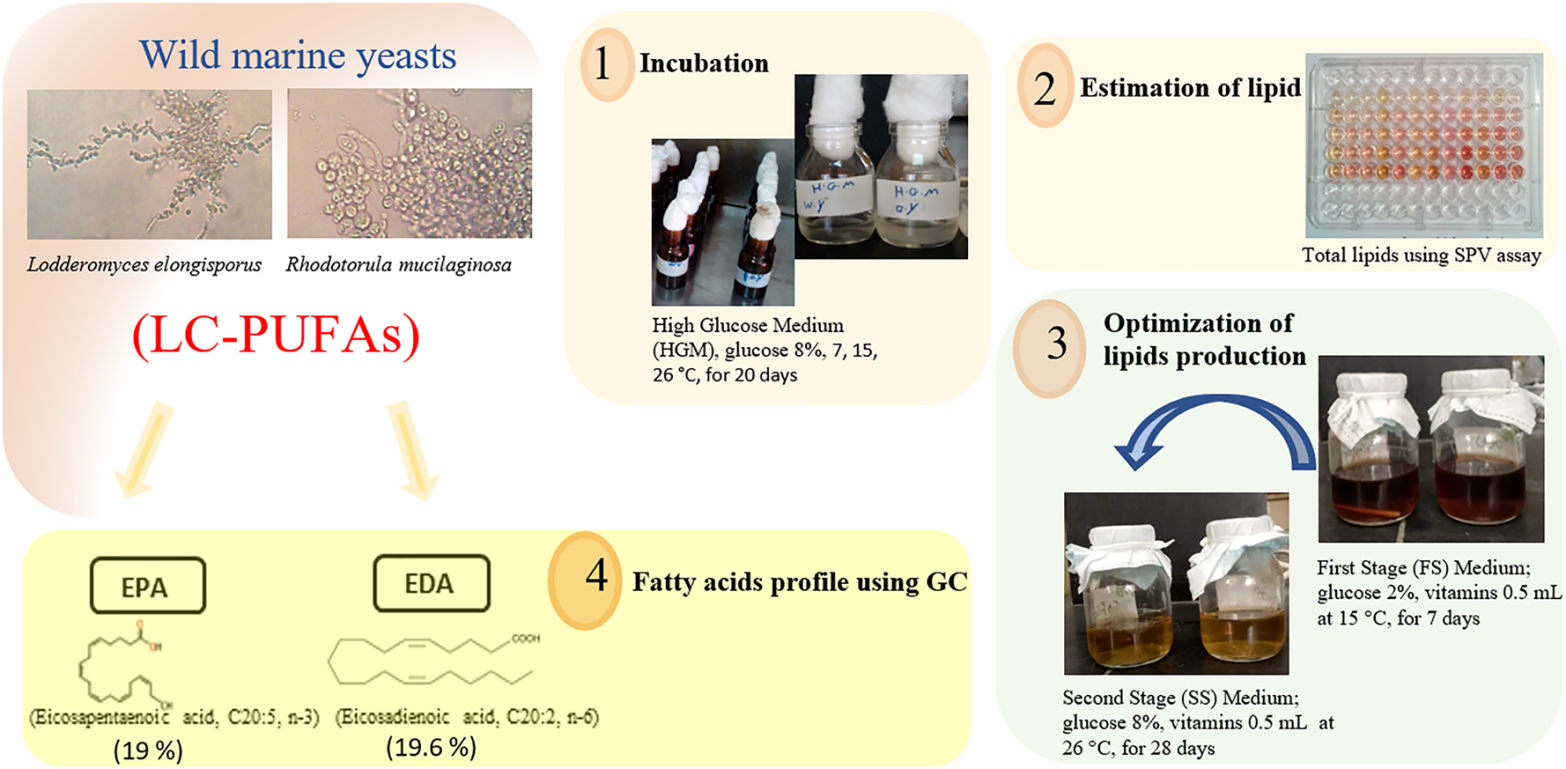

## Introduction

Omega-3 PUFAs, like eicosapentaenoic acid (EPA) and docosahexaenoic acid (DHA), are crucial in maintaining human health. They help reduce blood pressure, alleviate inflammation, and protect against chronic diseases such as heart disease, diabetes, and cancer. Also, it is highly recommended to regularly incorporate essential omega-3 PUFAs like Linoleic acid (LA) (n − 6) and α-Linolenic acid (ALA) (n − 3) into the daily diet since the human body cannot synthesize them due to the lack of essential enzymes like delta-12 and delta-15 desaturase (Saini and Keum 2018). However, challenges such as bad taste, fishy odor, stability issues, coextracted contaminants, and declining fish stocks worldwide raise awareness to explore alternatives to obtaining EPA and DHA rather than fish oils. Consequently, the natural production of omega-3 by marine microbes presents a promising avenue for this purpose. Producing omega-3-rich oils sustainably and mitigating the challenges associated with fish oils (Shah et al. 2022; Abbas et al. 2023).

Marine environments never fail to surprise researchers because they can harbor a wide variety of microorganisms that are capable of producing a variety of valuable compounds, such as lipids, enzymes, antibiotics, and therapeutic metabolites (Zaky et al. 2014; El-Baz et al. 2018; El-Far et al. 2021). Marine yeasts and their lipid contents as dietary supplements lack sufficient information in the context of lipid production. Adjusting growth conditions, such as carbon supply and other minerals, as well as pH and temperature, can significantly affect the total lipids content and the makeup of cellular fatty acids in a specific yeast strain (Elfeky et al. 2020).

Although most oleaginous yeasts develop triacylglycerides (TAGs) in response to nitrogen starvation (Donzella et al. 2019), alternative nutrient limitation techniques may be beneficial. For example, phosphorus and sulfur limitations allow lipid formation by *Rhodosporidium toruloides* mitigating, high nitrogen toxicity in low-cost substrates (Wu et al. 2011; Wang et al. 2018).

Additional evidence that nitrogen limitation during growth causes an increase in lipid accumulation was confirmed by Sitepu et al. (2013). *Cryptococcus curvatus*, on the other hand, is a remarkable exception; it can build TAGs before nitrogen depletion and even when undergoing logarithmic growth (Gong et al. 2015). As a result, lipid synthesis in nitrogen-rich substrates can be accelerated and the production time reduced, making them industrially applicable.

Different yeasts have been used to study the effects of nitrogen sources on total lipids and fatty acids (FAs) profiles, with different results. When *Cryptococcus albidus* was grown on different sources of nitrogen, the growth curves were almost the same (Hansson and Dostálek 1986). Compared to a variety of inorganic and organic compounds were evaluated as sole nitrogen sources to support the growth of *Rhodosporidium toruloides*, Evans and Ratledge evaluated urea as a sole nitrogen source for *R. toruloides* cultivation and reported it supported the highest levels of both biomass (18% w/w dry cell weight, DCW) and total lipids (52% w/w) (Evans and Ratledge 1984). Deeba and his team also examined the effects of various nitrogen compounds, including peptone, urea, yeast extract, and ammonium sulfate ((NH_4_)_2_SO_4_), on *R. toruloides* growth. Despite all nitrogen sources enabling cellular proliferation, significant differences in biomass and lipid production were observed. Yeast extract facilitated the maximum DCW of 10.2 g/L. However, the use of (NH_4_)_2_SO_4_ boosted the highest microbial lipid titer to 5.8 g/L, resulting in a lipid content of approximately 57% (w/w) DCW. These results indicate that different nitrogen sources can influence the carbon partitioning between biomass and lipid biosynthesis in *R. toruloides* (Deeba et al. 2021).

For non-oleaginous yeasts (Crabtree-positive yeasts), protein synthesis and development cease when the medium undergoes nitrogen depletion, and the extra carbon is transferred to build polysaccharides. Meanwhile, in oleaginous yeast species, the conversion of excess carbon is channeled into lipid bodies in the form of TAGs (Ageitos et al. 2011). In general, glucose inhibits the formation of mitochondria, and cells grown in high glucose concentrations have extremely poor respiratory activity, even under aerobic conditions (Kamihara and Nakamura 1984; de Alteriis et al. 2018). Hence, oleaginous yeasts (OY) help use high-sugar hydrolysates like cassava starch because they can quickly build up a large amount of cell mass and lipids (Li et al. 2010).

In addition, the significant effects of temperature variation as well as glucose concentration on FAs' composition were investigated in other studies (Rossi et al. 2009; Amaretti et al. 2010). Hence, the enhancement of growth temperature or glucose concentration could lead to an improvement in the FAs production (Salvador López et al. 2022).

*Rhodotorula* spp. are widely recognized oleaginous yeasts that have garnered significant attention in biofuel production and the synthesis of short-chain PUFAs (Viñarta et al. 2020; Maza et al. 2021). As an oleaginous yeast, it possesses the remarkable ability to accumulate lipids, making it a promising candidate for sustainable biofuel production. Additionally, *Rhodotorula mucilaginosa* has been extensively studied for its capacity to biosynthesize short-chain PUFAs, essential nutrients with various health benefits. The yeasts’ metabolic versatility and ability to efficiently convert carbon sources into valuable lipids and PUFA products have positioned them as useful organisms in biotechnological applications (Adel et al. 2021; Li et al. 2010 and Li et al. 2022). *Lodderomyces elongisporus*, a recently isolated strain, has emerged as a promising candidate in the production of PUFAs. In our previous published research paper and through meticulous experimentation and analysis, we have demonstrated the strain's capacity to efficiently accumulate lipids (54%) and synthesize short-chain PUFAs as Linoleic acid (22.67%) and α-Linolenic acid (7.47%) (Adel et al. 2021). *R. mucilaginosa* and *L. elongisporus* are oleaginous psychrophilic marine yeasts (Van der Walt 1966). Both strains accumulate 48–54% of lipids in a High-Glucose Basal Defatted Medium with C: N (8:1) at 15 °C (Adel et al. 2021).

Vitamins play a critical role in supporting the growth and metabolic processes of yeast; lack of biotin retards yeast growth and fermentation (Magdouli et al. 2020). Stambuk et al. (2009) reported that the amounts of unsaturated FAs in *S. cerevisiae* were decreased significantly in the presence of thiamine but were entirely avoided by the addition of pyridoxine with thiamine to the medium (Stambuk et al. 2009). Earlier studies suggest that thiamine is essential for the survival and growth of certain fungi, and it is also involved in the metabolism of glucose and fructose, as well as energy generation (Chung et al. 2009; Perli et al. 2020). In addition, adding pyridoxine would be essential to restore respiratory activity in high glucose medium (Nakamura et al. 1980; Evers et al. 2021). The long-term effects of vitamins on weight gain were studied by Pannia and his colleagues (Pannia et al. 2015). The researchers focused on the methyl group vitamins (i.e., folic acid, vitamin B12, and vitamin B6) and their functions within a high multivitamin diet. Vitamin B6 derivatives, like pyridoxal 5'-phosphate, were shown to stimulate adipogenesis and increase lipolysis in mouse 3T3-L1 adipocytes (Yanaka et al. 2011).

Furthermore, phospholipids biosynthesis requires methyl radicals, which are synthesized from methionine with the help of coenzymes B12 and B9 (Fidanza and Audisio 1982). Besides, the membrane structure of mitochondria and the production of lipids were not observed in pantothenate-deficient yeast cells (Furukawa and Kimura 1971). With the addition of pantothenic acid (vitamin B5) to deficient *S. cerevisiae*, unsaturated FAs, particularly palmitoleic acid, and oleic acid, were produced, and the rate of respiration in the defective cells was eventually recovered (Hosono and Aida 1974). It has also been shown that Orotic Acid (OA) stimulates lipogenesis (Jung et al. 2011).

Interestingly, two-stage fed-batch fermentation has been shown in previous studies to improve lipids production in yeast (Xie et al. 2017). Research into micro- and lab-scale fermentation eventually leads to the need for fermentation optimization (Xie 2012; Xie et al. 2015). Since PUFAs are made inside yeast cells, optimizing the process means first increasing biomass synthesis and then increasing PUFA production while stopping the formation of byproducts during the oleaginous phase. The use of two stages of fed-batch fermentation increases biomass and PUFA synthesis. First, yeast is grown on a nitrogen-rich carbohydrate substrate. Even though nitrogen is limited in the second stage, there are still enough carbohydrates. After using all the extra nitrogen in the medium, yeast cells stopped growing and stored lipids. Because strains have various genetic backgrounds, optimal conditions frequently differ (Zhu and Jackson 2015). Consequently, more improving work is likely to increase the performance of the newly selected strains. For the aforementioned reasons, the objective of this study was to investigate the influence of different incubation temperatures and the effect of the depletion in nitrogen supply on lipids production by the two wild marine yeasts (*R. mucilaginosa* and *L. elongisporus*), using the two-stage batch fermentation bioprocess to boost the cellular biomass and PUFAs synthesis.

## Materials and methods

### Chemicals and media

Anhydrous D-glucose, methyl alcohol, and phosphoric acid were purchased from Alamia Chemicals, El-Nasr Pharmaceutical Chemicals Co., and El Gomhouria Co. in Egypt. Techno Pharmchem and Loba Chemie (India) sold yeast extract, peptone, and vanillin, respectively. Sri-Parme was a supplier of agar. Chloroform was obtained from Fisher Scientific, UK. Other locally obtained analytical reagent grade chemicals and reagents were used.

The medium Yeast Extract–Peptone–Dextrose (YPD) was used for the seed culture and maintenance of yeast isolates with the composition (g/L); yeast extract 10, peptone 20, glucose 20, agar 20, pH 5.5 ± 0.2. High Glucose Medium (HGM) was used as a minimal medium for lipid production by marine yeast isolates in different incubation temperatures with the composition (g/L); glucose 80, KH_2_PO_4_ 6.3, 27 K_2_HPO_4_ 27, pH 6.8. First Stage (FS) growth medium was used for biomass production in a two-stage fermentation system with the following composition (g/L): yeast extract 10, peptone 20, glucose 20, MgCl_2_ 1, ZnSO_4_.7H_2_O 0.02, vitamin supplements 0.5 mL, pH 5.8 ± 0.2. Second Stage (SS) fermentation medium or High glucose medium with vitamins (HGM + VIT) was used for lipids production in the two-stage fermentation system with the following composition (g/L); glucose 80, KH_2_PO_4_ 6.3, K_2_HPO_4_ 27, MgCl_2_ 1, ZnSO_4_.7H_2_O 0.02, and vitamins (0.5 mL, pH 5.8). Vitamin supplements were modified according to Adel et al. (2021). These supplements were group of Vitamins B and Orotic acid used for lipid production with the following composition (mg/mL); B_1_ (Thiamine) 2.5, B_3_ (Nicotinamide) 10, B_5_ (Pantothenic acid) 3, B_6_ (Pyridoxine hydrochloride) 2, B_9_ (Folate) 0.5, B_12_ (Cyanocobalamine) 1.25, and Orotic acid 5.

### Yeast strains identification

The two marine isolates, *R. mucilaginosa* and *L. elongisporus* were isolated from the Red and Mediterranean Seas, Egypt and identified by MALDI-TOF analysis in our laboratory and preserved in GEBRI Microbiological Culture Center (University of Sadat City, Egypt). Both strains were stored in YPD slants at 4 °C and subcultured every two weeks (Adel et al. 2021). The identity of the tested yeast isolates was confirmed molecularly from the sequence of the ITS region. The genomic DNA of the yeasts was extracted by the CTAB method (El-Sayed et al. 2015, 2022; Abdel-Fatah et al. 2021), and used as PCR template, with the primer set ITS4 5′-GGAAGTAAAAGTCGTAACAAGG-3′ and ITS5 5′-TCCTCCGCTTATTGATAT-GC-3′. The PCR reaction contains 10 μl of 2 × PCR master mixture (Cat. # 25027), 1 μl gDNA, 1 μl of each primer (10 pmol/μl), in total volume 20 μl with sterile distilled water. The PCR was programmed to initial denaturation at 94 °C for 4 min, 35 cycles at 94 °C for 30 s, 55 °C for 10 s, and 72 °C for 30 s, in addition to final extension at 72 °C for 4 min. The amplicons were analyzed by 2.0% agarose gel in TBE buffer, sequenced by Applied Biosystems Sequencer, HiSQV Bases. The obtained sequences were non-redundantly BLAST searched, aligned by the Clustal W muscle algorithm (Tamura et al. 2011), and the phylogenetic analysis was constructed with the neighbor-joining method of 100 bootstrap replications (Edgar 2004).

### Lipids production and growth at different temperatures

For investigating the influence of diverse temperature regimens on lipids synthesis over an extended incubation period, two seed cultures of the yeast isolates were used to inoculate High Glucose Medium (HGM) at a 1:10 [v/v] ratio in three experimental groups. The cultures were incubated at 7 °C, 15 °C and 26 °C in an orbital shaker (New Brunswick, CA) set to 150 rpm for 480 h (20 days). At five-day intervals, samples were withdrawn by removing three containers from each temperature. The yeast cells were harvested by centrifugation at 5000x*g* at 4 °C, washed twice with sterile saline solution, and then resuspended to a volume of 1 mL prior to storage at − 20 °C for subsequent analysis.

### Two-stage batch fermentation

First Stage (FS) Growth Media were prepared by transferring a single colony grown on solid YPD into a 500 mL Erlenmeyer flask with 200 mL of FS Media, capped with cotton plug and incubated at 15 °C and 150 rpm for 168 h (7 days). Second-Stage (SS) Fermentation Media (HGM + VIT): Yeast cultures were cultivated in 500 mL Erlenmeyer flasks containing 200 mL of SS medium and capped with breathable cotton filter masks (> 99% Bacterial Filtration Efficiency (BFE); > 99.5% Particles Filtration Efficiency, PFE). SS medium was inoculated with 20 mL of FS medium (1:10 [v/v]), then incubated at 26 °C for 672 h (28 days) at 150 rpm. For lipids analysis, the yeast cells were harvested via centrifugation at 5000x*g* and 4 °C, followed by twice washing the cell pellets with sterile saline solution then re-suspended to a 1 mL volume prior to storage at − 20 °C. Concurrently, the dry cell weight (DCW, g/L) of each sample was determined by placing samples at 80 °C until constant weight. At the end of the incubation period, reducing sugars residues were measured using the 3,5-Dinitrosalicylic acid method (Zhao et al. 2008).

### Sulfo-phospho-vanillin (SPV) assay for lipids estimation

Lipids were measured using a modified SPV assay based on Mishra et al. (2014) method, and the standard lipid stocks were prepared according to Cheng et al. (2011) and Adel et al. (2021) modified method. 10 mg/mL of pre-washed yeast cells were transferred to 96-well microplate at different aliquots, diluted to 50 μL with distilled water. Sulfuric acid (100 μL) was added and mixed intensively, then incubated for 20 min at 90 °C. The reaction mixtures were rapidly iced-cooled, and the initial vanillin background absorbance was measured at *λ*_570_. 100 μL of SPV reagent (0.2 mg vanillin/mL in 17% phosphoric acid) was added, incubated for 10 min at 25 °C in the dark, and a post-vanillin absorbance was measured. The final SPV response was defined as the difference in absorbance of post-vanillin and initial-vanillin measured spectrophotometrically at *λ*_570_ (Adel et al. 2021).

### Extraction of lipids

For lipids extraction, yeast biomass suspension was disrupted based on the modified method described by Byreddy et al. (2015). The yeast biomass (500 mg) was suspended in 10 mL of 10% NaCl solution, vortexed for 2 min, and incubated for 48 h at room temperature. The shearing force was then produced by re-pipetting for 60 min with 5-min rest intervals using a 20-mL glass syringe with its needle. A four-step procedure for lipid extraction was used, as described by Axelsson and Gentili (2014). 230–300 mg samples of yeast cell pellets were re-suspended in 0.5 mL of 0.73% NaCl solution and mixed with 10 mL chloroform: methanol mixture (1:1) for 30 min. The centrifuged pellet was extracted again with 3 mL of the same solvent (chloroform: methanol; 1:2) and chloroform: methanol: 0.73% NaCl (60:30:4.5). The solvents were evaporated under a stream of nitrogen gas. The dry residue was employed for further purification.

### Methylation of fatty acids and gas–liquid chromatography

FA composition of the extracted lipids was determined according to the method of Jøstensen and Landfald (1997). Derivatization to fatty acid methyl esters (FAMEs) (via methanolic KOH) was performed as described by Adel et al. (2021) at the Regional Center for Food and Feed, Cairo, Egypt. FAs composition was determined using PerkinElmer (Waltham, MA) Clarus-580 GLC equipped with HP88 capillary column (30 m × 0.25 mm i.d, 0.20 μm film thickness) and FID, and a GLC-461 (NuChek Prep, Inc., Minnesota, USA) as a reference standard for the determination of C_12_–C_24_ FAs.

### Statistical analysis

Every experiment was run three times. The means ± standard deviation were displayed for the results. Using SPSS (Version 17), a two-way ANOVA was conducted to examine the effect of temperature and time on lipid synthesis, with significant differences determined at *P* ≤ *0.05*.

## Results

### Molecular identification of yeast strains

**Fig. 1:**
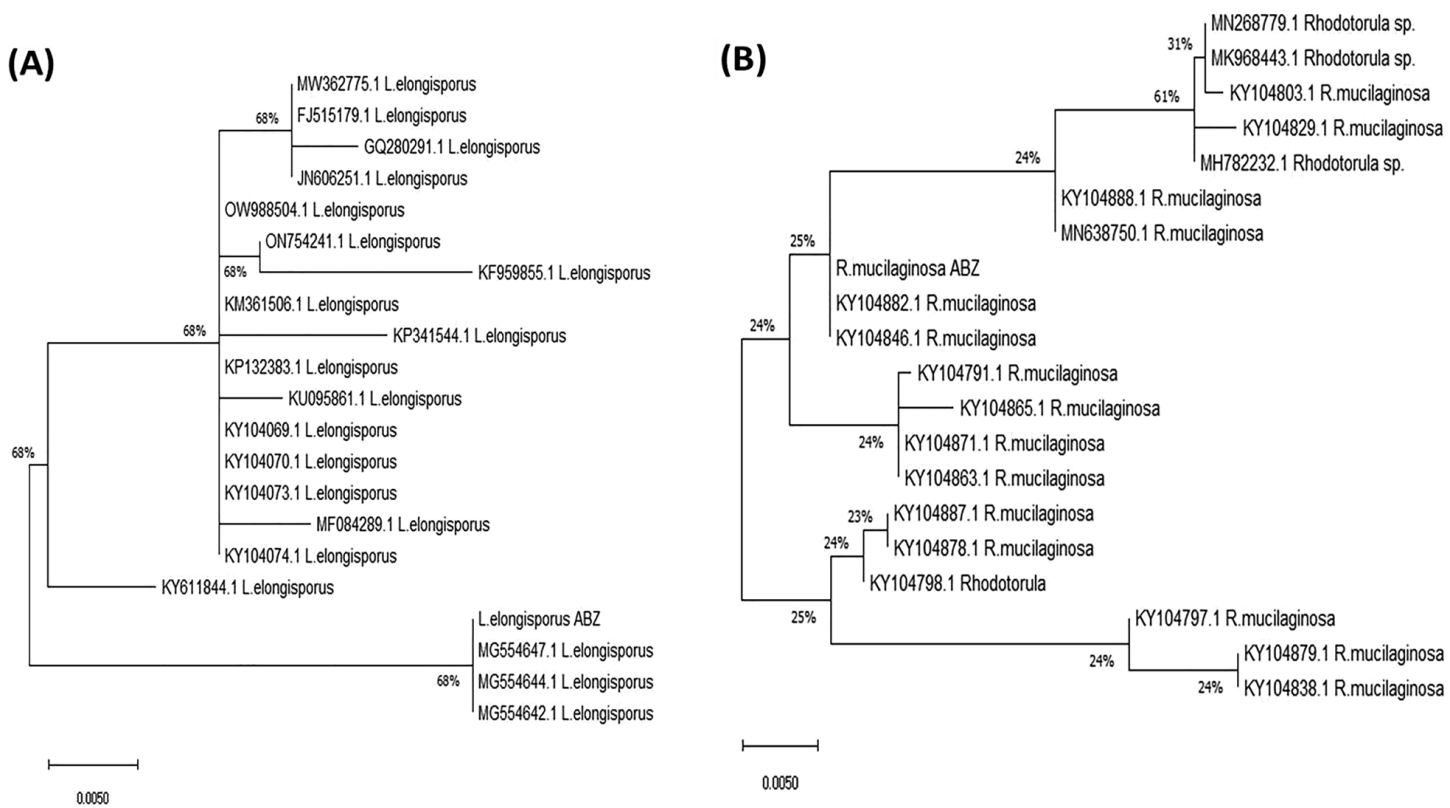
Molecular identification of *Lodderomyces elongisporus* and *Rhodotorula mucilaginosa* based on the sequence of the ITS region. The phylogenetic relatedness of *L. elongisporus* (**A**) and *R. mucilaginosa* (**B**) by the Maximum Likelihood method

The marine yeasts isolated from the Red and Mediterranean Sea, Egypt were identified by MALDI-TOF as *R. mucilaginosa* and *L. elongisporus* (Adel et al. 2021)*.* The biochemical identified yeasts were molecular confirmed by their ITS sequences. The genomic DNA of the yeasts were used as PCR template, the amplicons for the two yeasts were resolved by about 550–600 bp. The amplicons were sequenced, and the retrieved sequences were non-redundantly BLAST searched on the NCBI database, displaying 99.5% similarity with the ITS sequence of *R. mucilaginosa* and *L. elongisporus.* The yeasts *R. mucilaginosa* and *L. elongisporus* were deposited at the Genbank with accession numbers OR975650 and OR975649, respectively. From the phylogenetic analysis of ITS sequences of *R. mucilaginosa*, the ITS sequence of this isolate had about 99% similarity with various isolates *R. mucilaginosa*, with accession # KY104882.1, KY104846.1, KY104791.1, KY104865.1, KY104871.1, KY104863.1, KY104887.1, KY104798.1, MN638750.1, KY104888.1, and KY104797.1, with E-value zero and 99% query coverage (Fig. 1). As well as, the ITS sequence of *L. elongisporus* displayed 98% similarity ITS sequence of *L. elongisporus* with accession numbers MG554647.1, MG554644.1, MG554642.1, KY611844.1, MF084289.1, KY104073.1, KY104070.1, KY104069.1, KP132383.1, KP341544.1 and KM361506.1 with E-values zero and 98% query coverage (Fig. 1). So, from the biochemical and molecular analysis, the yeasts were confirmed as *R. mucilaginosa* and *L. elongisporus.*

### Yeast growth at different temperatures in HGM medium

**Fig. 2:**
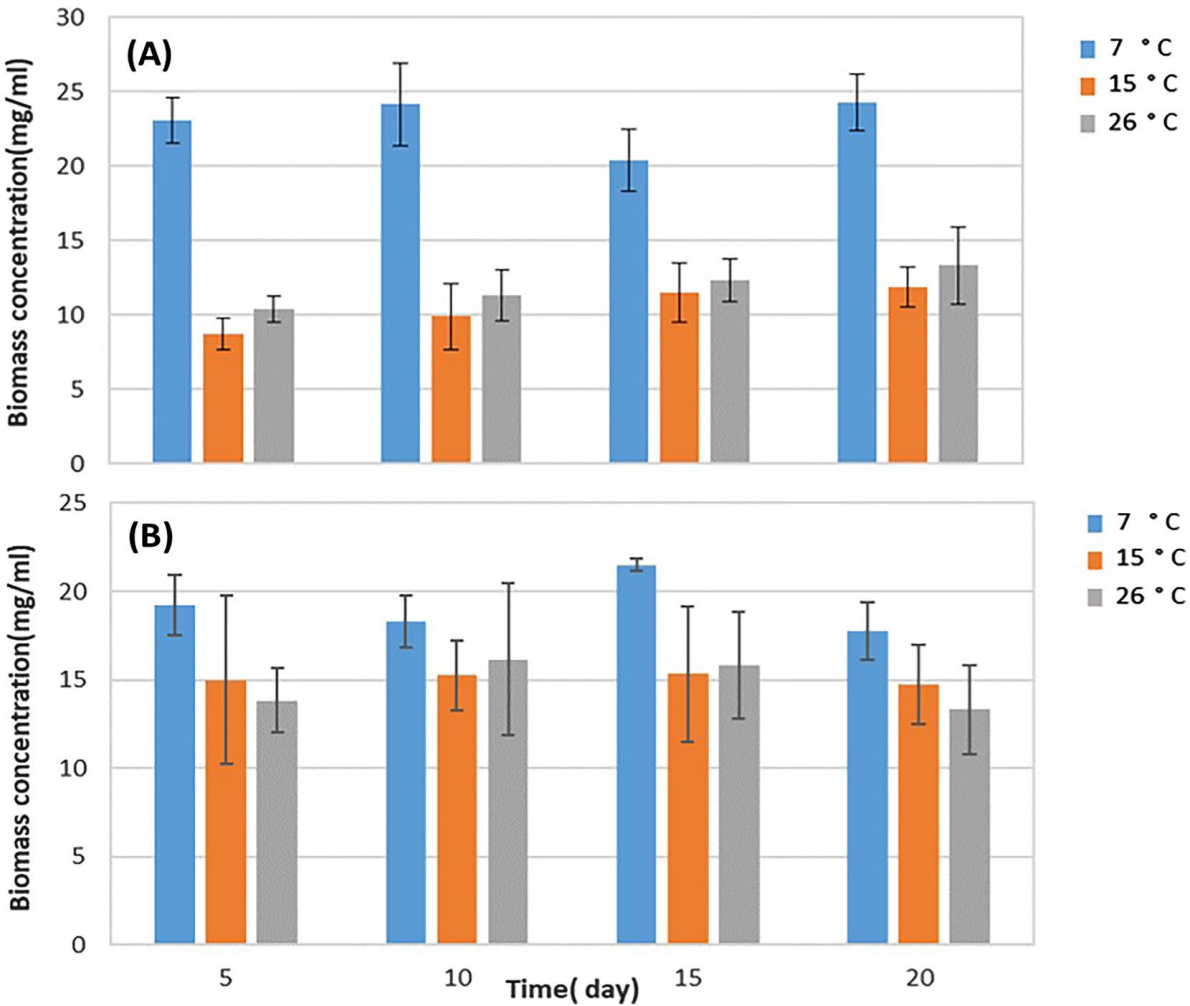
Biomass concentration (g/mL) of *L. elongisporus* (**A**) and *R. mucilaginosa* (**B**) after incubation at 7, 15 and 26 °C in HGM (average ± SD)

The oleaginous yeast strains *R. mucilaginosa* and *L. elongisporus* were cultivated in HGM medium with a carbon to nitrogen ratio of 8:0 to study the effect of temperature on biomass production. Maximum dry cell weight (DCW) for *R. mucilaginosa* was achieved after 15 days of cultivation at 7 °C, reaching 21.5 ± 2.4 mg/mL. Similarly, *L. elongisporus* attained its highest DCW of 24.3 ± 0.9 mg/mL after 20 days at the same temperature. In contrast, incubation of the yeasts at elevated temperatures corresponded to significantly lower biomass yields. Specifically, *R. mucilaginosa* cultured at 26 °C for 20 days produced a DCW of only 13.3 ± 4.2 mg/mL. Similarly, L. elongisporus incubated at 15 °C attained a maximum DCW of just 8.8 ± 1.02 mg/mL after 5 days. Nonetheless, there was no significant difference among measured values over time, as shown in Fig. 2. To demonstrate the effectiveness of the microplate SPV approach for lipids measurement of intact yeast cells cultivated in HGM at, 7 °C, 15 °C and 26 °C both yeast isolates' biomass was collected and washed, and the lipids content of a known mass of cells (10 mg/mL) was measured at four concentrations. Absorbance versus simulated liquid culture volume showed a linear relationship with a significant linear correlation (R^2^ > 0.95) despite some exceptions (Table 1). The lipid content was determined by converting absorbance to mass using known standard lipids (Fish, Flax seed, and Coconut oils) described by Adel et al. 2021. The linearity of the test was verified by plotting detected lipid versus added biomass for each isolate (Table 1). The response factor ranged from 0.2318 to 2.5909, and the correlations between the measured lipids concentrations and the yeast cells were greater than 0.85 overall (Table 1), normalized to standard curves. Experiments with both yeast isolates were chosen from sets incubated at 7 °C on the 15th day and at 15 °C and 26 °C on the 20th day due to their significantly high response factors (≥ 0.7) as compared to the standard curves (Table 1) to further analysis.

**Table 1:** Evaluation of lipid synthesis using the SPV test in two yeast isolates cultured for 20 days at three different temperatures (7, 15, and 26 °C) in HGM

Incubation temperature	Yeast	Days	Slope 1	Slope 2	Slope 3	Correlation R^2^
(a) 7 °C	*R. mucilaginosa*	5	0.6098	0.3084	0.0161	0.9046
		10	1.4432	0.7299	0.0381	0.9866
		15	1.4356	0.7261	0.0379	0.9996
		20	0.5947	0.3004	0.0157	0.9431
	*L. elongisporus*	5	0.4773	0.2414	0.0126	0.961
		10	1.9621	0.9923	0.0518	0.9587
		15	1.3485	0.682	0.0356	0.9166
		20	0.4773	0.2414	0.0126	0.9041
(b) 15 °C	*R. mucilaginosa*	5	0.6553	0.3314	0.0173	0.9958
		10	0.7273	0.3678	0.0192	0.9799
		15	1.6061	0.8123	0.0424	0.9232
		20	1.4735	0.7452	0.0389	0.9892
	*L. elongisporus*	5	0.8258	0.4176	0.0218	0.8594
		10	0.9015	0.4559	0.0238	0.9776
		15	1.3258	0.6705	0.035	0.9838
		20	1.4356	0.7261	0.0379	0.9378
(c) 26 °C	*R. mucilaginosa*	5	0.7765	0.3927	0.0205	0.9453
		10	2.5909	1.3103	0.0684	0.9588
		15	1.1591	0.5862	0.0306	0.9717
		20	1.7045	0.8621	0.045	0.9843
	*L. elongisporus*	5	0.6477	0.3276	0.0171	0.9601
		10	0.4583	0.2318	0.0121	0.9936
		15	1.1023	0.5575	0.0291	0.9395
		20	1.1098	0.5613	0.0293	0.9902

Fish oil and Flax seed oil were used as external standards for the conversion of absorbance units to µg of lipids. Slope 1; from linear equation using Flax seed oil as an external standard. Slope 2; from linear equation using Fish oil as an external standard. Slope 3; from linear equation of SPV-microassay (absorbance measured at 570 nm)

### Optimization stage using two-stage batch fermentation

Yeast isolates were initially screened using the SPV assay to evaluate relative lipid accumulation when cultured in HGM. Strains displaying positive results in the SPV assay, indicating enhanced lipid droplet formation, were selected for lipid extraction, followed by gas chromatography (GC) analysis. The GC profiles (Table 2) revealed that *R. mucilaginosa* and *L. elongisporus* cultured at 26 °C exhibited unique fatty acid compositions when compared to other temperatures, dominated by monounsaturated fatty acids (MUFAs) such as palmitoleic acid, cis-7-hexadecenoic acid, and vaccinic acid. Additionally, these two strains uniquely produced of polyunsaturated fatty acids α-linolenic acid and stearidonic acid, as well as long-chain fatty acids like gadoleic acid, when cultured at 26 °C. Due to this promising fatty acid profile characterized by specific MUFA and polyunsaturated fatty acid production exclusively at 26 °C, this temperature was carried forward for the optimization stage. *R. mucilaginosa* and *L. elongisporus* were cultured at 26 °C in HGM + VIT (SS fermentation media). The collected isolates were cultured in HGM as well as HGM + VIT, which previously underwent lipid extraction and were evaluated by GC to quantify and compare the FAME profiles and total lipid content between the different fermentation conditions (Table 2). This experimental workflow provided a preliminary screening of isolates via a qualitative lipid staining method and statistical comparison across varying culture temperatures (Table 1), followed by quantitative lipid analysis (Table 2).

**Table 2:** Estimation of the fatty acid contents (%) of *L. elongisporus* and *R. mucilaginosa* following incubation with HGM and HGM + VIT

Fatty acids	Name	*L. elongisporus*			*R. mucilaginosa*			*L. elongisporus*	*R. mucilaginosa*
		HGM						HGM + VIT	
		7 °C	15 °C	26 °C	7 °C	15 °C	26 °C	26 °C	26 °C
C12:0	Lauric acid	–	4.46	2.87	–	–	–	1.11	–
C13:0	Tridecanoic acid	–	–	–	–	9.12	1.01	0.87	–
C14:0	Myristic acid	–	–	7.01	–	4.81	5.52	0.43	–
C15:0	Pentadecanoic acid	–	–	0.80	–	–	0.79	–	–
C16:0	Palmitic acid	16.20	31.20	24	20.21	37.1	23.86	10.4	–
C16:4 ω1	6,9,12,15 hexadecatetraenoic acid	–	–	–	–	–	–	0.44	3.83
C16:1 ω7	Palmitolic acid	–	–	1.45	–	–	–	1.33	–
C16:1 ω9	cis-7 Hexadecenoic acid	–	–	–	–	–	2.27	–	–
C16:4 ω3	Hexadeca tetraenoic acid	–	–	–	–	–	–	0.44	–
C16:3 ω4	Hexadeca trienoic acid	–	–	–	–	–	–	5.30	–
C17:0	Heptadecanoic acid	–	–	0.77	–	–	1.48	3.76	2.78
C18:0	Stearic acid	–	14.35	9.89	11.1	29.6	7.76	7.70	11.0
C18:1 ω7	Vaccinic acid	–	–	3.12	–	–	3.14	–	–
C18:1 ω9	Oleic acid	48.86	25	30.26	29.70	13.3	32.04	40.6	6.24
C18:2 ω6	Linoleic acid (LA)	34.94	20.18	13.82	32.80	10.8	18.35	8.33	5.25
C18:3 ω3	α-Linolenic acid (ALA)	–	–	1.59	–	–	2.80	0.48	–
C18:4 ω3	Stearidonic acid (SDA)	–	–	0.96	–	–	–	–	–
C20:1 ω11	Gadoleic acid; cis-9-Eicosenoic acid	–	–	1.41	–	–	0.96	–	–
C20:5 ω3	Eicosapentaenoic acid (EPA)	–	–	–	–	–	–	19.0	–
C20:2 ω6	Eicosadienoic acid (EDA)	–	–	–	–	–	–	–	19.6
C22:0	Behenic acid	–	–	–	–	–	–	–	29.6
C22:2	Docosadienoic acid	–	–	–	–	–	–	0.43	–
C22:2 ω6	cis-13,16-Docosadienoic acid	–	–	–	–	–	–	0.35	3.78
NA	Non-Identified FA	–	–	–	6.19	–	0.02	0.01	0.14
Total SFA		16.2	50.01	45.34	31.31	80.63	40.42	24.27	43.38
Total MUFA		48.86	25	36.24	29.7	13.3	38.41	41.93	6.24
Total PUFAs		34.94	20.18	16.37	32.8	10.8	21.15	34.33	32.46
Total UNSFA		83.8	45.18	52.61	62.5	24.1	59.56	76.26	38.7

*SFA* saturated fatty acid, *MUFA* monounsaturated fatty acid, *PUFAs* polyunsaturated fatty acids, *UNSFA* unsaturated fatty acid, *HGM* High glucose medium, *HGM + VIT* High glucose medium + vitamin supplement

At the first stage, after incubating for 168 h with a first-stage (FS) growth medium containing 2% glucose and 0.5 mL of vitamin supplements, moderate increases in DCW were observed for both *L. elongisporus* and *R. mucilaginosa* as compared to their growth in YPD medium (containing 1% glucose) at 15 °C. Specifically, the DCW of *L. elongisporus* increased from 13.4 to 19.3 mg/mL when cultured in the FS medium, representing a modest 44% rise. Similarly, *R. mucilaginosa* exhibited a DCW of 25.8 mg/mL when grown in the FS medium, marking an incremental increase of 42% over its DCW of 18.2 mg/mL in standard YPD medium under the same temperature conditions. These results indicate that supplementing the growth environment with additional glucose and vitamins led to small but measurable enhancements in biomass accumulation over a cultivation period for both yeast strains.

### Comparison of fatty acids composition

Lipids profiles of *R. mucilaginosa* and *L. elongisporus* (Table 2) mainly consisted of long-chain FAs with between sixteen and eighteen carbon atoms. These findings revealed that the distribution of several FAs, including C18:1 (oleic acid), C16:0 (palmitic acid), C18:0 (stearic acid), and C18:3 (linolenic acid), were almost dominant throughout the process. *R. mucilaginosa* and *L. elongisporus* had the highest values of 32.80% and 34.94% (C18:2; linoleic acid) at 7 °C, respectively, but the lowest values were obtained at 26 °C.

When *R. mucilaginosa* was cultivated in HGM + VIT under the same conditions as *L. elongisporus*, the relative concentration of oleic acid (C18:1) was significantly decreased. Monosaturated FAs were found only at 26 °C in HGM, such as gadoleic acid and vaccinic acid. Palmitolic acid (C16:1, ω7) and cis-7 Hexadecenoic acid (C16:1, ω 9) were also detected in HGM + VIT and HGM, respectively. HGM + VIT, showed unusual C16 PUFAs (C16:4, ω 1; 6,9,12,15-hexadecatetraenoic acid), C16:3 (ω4; hexadeca tertaenoic acid), and C16:3 (ω4; hexadeca trienoic acid) in relatively low concentrations (< 6%). Therefore, there was a substantial correlation between C16 UNSFAs and the temperature rise. Furthermore, C18 PUFAs (other than LA) were found in both yeasts at 26 °C, including C18:3 ω 3 (α -Linolenic acid; ALA) and C18:4 ω 3 (Stearidonic acid; SDA), albeit in trace amounts (< 3%).

The incubation at 26 °C is optimal for producing of long-chain fatty acids (LCFAs) (C20 and C22). Interestingly, *L. elongisporus* generated a significant quantity of Eicosapentaenoic acid (EPA) (19%) after being incubated for 28 days in the two-stage HGM + VIT. Simultaneously, *R. mucilaginosa* stored 19.6% Eicosadienoic acid (EDA) in the same conditions. Both yeast isolates maintained stable and maximal levels of total PUFAs (34.94, 32.8%) at 7 °C with HGM and (34.33, 32.46%) at 26 °C with HGM + VIT, respectively (Table 2). On the other hand, *L. elongisporus* and *R. mucilaginosa* incubated at 15 °C showed the highest levels of 50 and 80.6% of total SFAs, respectively, in HGM.

## Discussion

In our previous study, we observed an increase in FAs unsaturation degree for both yeast strains with a decreasing incubation temperature (7 °C and 15 °C) in basal defatted medium (BDM) (3% glucose), mainly in linoleic acid (LA) and α-linolenic acid (α-LA). In contrast, the opposite trend was observed with the increasing glucose concentration from 3 to 8% at 7 °C in High-Glucose Basal Defatted Medium (HG-BDM) (8% glucose), mainly in the absence of α-linolenic acid (α-LA). Meanwhile, HG-BDM positively affected total lipid production, reaching its maximum of 48% and 54% by *R. mucilaginosa* and *L. elongisporus*, respectively, at 15 °C (Adel et al. 2021).

Earlier research has demonstrated that the temperature of incubation and the concentration of glucose significantly impact the fatty acid (FA) composition of *Rhodotorula* species. For example, unsaturated FAs increase dramatically when the glucose concentration is raised from 2 to 10%. While oleic acid (OA) and linoleic acid (LA) more than doubled, alpha-linolenic acid (ALA) jumped from 1.7 to 6.21%. That tendency towards increased FA unsaturation as glucose levels rise at 15 °C was reported by Gupta et al. (2012). In the opposite direction, the FA profile of *Rhodotorula glacialis* was impacted by growth temperature and glucose concentration, with reduced fatty acid unsaturation occurring at higher temperatures or glucose concentrations (Amaretti et al. 2010). Consequentially, in the current study, we continued what we started to examine the effect of high glucose concentration at different growth temperatures on total biomass and productivity by the tested marine strains.

The obtained data revealed that lowering the temperature had a significant influence on boosting total lipids synthesis for both yeast isolates, as shown in Table 3. At 7 °C, HGM demonstrated a high theoretical lipid output of 0.16–0.17 (Y_lipid/glucose_) for ingested glucose. In comparison, relatively low constant values (0.07—0.08) were observed in the same medium at 15 °C and 26 °C (Table 3). Earlier investigations have also identified this pattern (Granger et al. 1993; Amaretti et al. 2010). Despite raising the temperature to 26 °C, HGM + VIT was second in high lipid production (0.14–0.18), compared to the comparatively low lipid yield (0.07–0.08) in HGM at the same temperature. The inclusion of vitamins and minerals as part of the two-stage fermentation culture method seems to have mitigated the impact of high temperature.

**Table 3:** The influence of temperature on the growth and lipid synthesis of *R. mucilaginosa* and *L. elongisporus*

Organism	Temp. (°C)	Day	Medium	Y_L/s_ (mg/g)	Y_L/x_ (mg/mg)	Y_x/s_ (mg/g)	Biomass (mg/mL)	Lipids (mg/mL)
*R. mucilaginosa*	7 °C	15	HGM	0.16	0.58	0.27	21.5	12.4
	15 °C	20	HGM	0.07	0.38	0.18	14.7	5.61
	26 °C	20	HGM	0.07	0.42	0.17	13.3	5.59
	26 °C	28	HGM + VIT	0.14	0.49	0.28	22.6	11.1
*L. elongisporus*	7 °C	15	HGM	0.17	0.66	0.26	20.4	13.4
	15 °C	20	HGM	0.06	0.37	0.15	11.9	4.41
	26 °C	20	HGM	0.08	0.48	0.17	13.3	6.39
	26 °C	28	HGM + VIT	0.18	0.53	0.34	27	14.2

*R. mucilaginosa* and *L. elongisporus* were cultured at different temperatures in HGM and HGM + VIT containing 80 g/L glucose. the ultimate concentrations of biomass and lipids, Y_X/S_, Y _L/S_, and Y_L/X_, are biomass/glucose, lipid/glucose, and lipid/biomass yield coefficients, respectively, these coefficients altered with growing temperatures

Vitamins significantly influence yeast growth, metabolic processes, and lipogenesis. The lack of biotin hinders yeast growth and fermentation (Magdouli et al. 2020), while thiamine decreases unsaturated fatty acid levels in *S. cerevisiae*. However, pyridoxine and thiamine can eliminate this effect (Stambuk et al. 2009). In addition, thiamine is crucial for fungus growth, survival, energy production, and glucose and fructose metabolism (Perli et al. 2020). Adding pyridoxine can restore respiratory activity in a high-glucose medium (Evers et al. 2021).

Pannia et al. (2015) investigated the impact of micronutrients on weight gain, focusing on the functions of methyl group vitamins (folic acid, vitamin B12, and vitamin B6) in a multivitamin-rich diet. Vitamin B6 derivatives promote lipolysis and stimulate adipogenesis (Pannia et al. 2015). Furthermore, methyl radicals are generated from methionine via coenzymes B12 and B9.

Pantothenate-deficient yeast cells cannot produce lipids and exhibit mitochondria without a membrane structure. When pantothenic acid (vitamin B5) is added, unsaturated fatty acids, specifically palmitoleic acid, and oleic acid, are generated in deficient *S. cerevisiae*, restoring respiration rates (Hosono and Aida 1974). Also, orotic acid (OA) has been shown to stimulate lipogenesis, yet its mechanism is obscure (Jung et al. 2011).

Moreover, magnesium and zinc are crucial for the survival of brewing yeast because they decrease cell death caused by ethanol stress and enhance the yeast's ability to tolerate stress (Walker 2000; De Nicola and Walker 2011). *Rhodotorula toruloides* can produce PUFAs when specific trace minerals are added to growth media. When grown on media enhanced with Mg^2+^, eicosatrienoic acid (ETA/C20:3) accounts for 0.03% of total fatty acids (TFA). Cu^2+^ supplementation produces docosahexaenoic acid (DHA/C22:6) with 0.05% TFA. γ-linolenic acid (GLA/C18:3) levels increase from 0.07% without metal addition to 0.22%, 0.20%, and 0.12% of TFA, respectively, with Zn^2+^, Fe^2+^, and Cu^2+^ supplementation (Saini et al. 2023).

The effect of temperature on DCW shows at 7 °C and 26 °C, increasing in DCW by 21.50 and 22.6 with HGM and 20.42 and 27.00 with HGM + VIT, respectively, even though cell proliferation halted in both culture modes due to nitrogen depletion. These findings might be due to the higher lipid content of aged fatty yeast cells rather than their growing biomass (Table 3). When cultivated in a two-stage fermentation culture mode, *L. elongisporus* surpasses *R. mucilaginosa* in total lipids accumulation (Table 3), with 14.2 mg/mL total lipids, and this is the first research dealing with lipids production by *L. elongisporus*. Since the first discovery of *L. elongisporus* from concentrated orange juice in 1952 and renamed by van der Walt (Kurtzman and Suzuki 2010; Van der Walt 1966a), their uses in industrial biotechnology are limited to fermenting Luzhou-flavor liquor (Ling et al. 2017), Petroleum-degradation (Ma et al. 2015) and lipase production (Wang et al. 2007). However, several previous studies have been on well-known *Rhodotorula* spp. in lipids synthesis, particularly for biodiesel production (Kot et al. 2016; Viñarta et al. 2020).

Furthermore, Gupta et al. (2012) suggested that the marine-derived *R. mucilaginosa* AMCQ8A might be a source of lipids (65% of total biomass), including omega-3 FAs (6–7%). Moreover, Sitepu et al. (2013) reported that several yeast strains were subjected to different culture conditions. Culture A involved a three-day cultivation period, Culture B involved a five-day cultivation period, and Culture C involved a five-day cultivation period with a transition from a low-nitrogen medium to a nitrogen-free medium with a high glucose content of 120 g/L. Out of the 69 oleaginous yeasts (OY) studied, 34 exhibited the most increased lipids content in Culture C, confirming that lipids accumulation in OY is promoted by nitrogen deprivation (Sitepu et al. 2013).

Nitrogen limitation can induce lipid accumulation in OY by limiting protein synthesis and reducing the demand for amino acid and nucleic acid precursors. The tricarboxylic acid (TCA) cycle becomes less active due to decreased glutamate production and alpha-ketoglutarate, a major carbon entry point into the TCA cycle. This reduces the production of intermediates like oxaloacetate, which are required for amino acid and nucleotide biosynthesis. Excess carbon is produced as acetyl-CoA through glycolysis and pyruvate dehydrogenase reaction, which is diverted from the TCA cycle to fatty acid synthesis via fatty acid synthase. Nitrogen limitation generally shifts carbon flux from energy production and biosynthesis towards storage as neutral lipids like triacylglycerols (Zhu et al. 2012). The nitrogen limitation approach is a well-established method for inducing lipogenesis in many oleaginous microbes (Zhu et al. 2012; Donzella et al. 2019). Sulfate limitation was found to promote intracellular lipid accumulation in *R. toruloides* up to 58.3% of cellular content, by cultivating the yeast in a medium with an initial high carbon-to-sulfur (C/S) molar ratio of 46,750, sulfate was depleted earlier, triggering a metabolic shift toward lipid biosynthesis (Wu et al. 2011). Sulfate needed for synthesis of sulfur-containing amino acids like cysteine and methionine, so, sulfate depletion reduces the carbon flux into central metabolic pathways, causing cells to divert excess carbon from glucose sources into lipid biosynthesis. Unlike the nitrogen limitation approach, phosphorus limitation offers a more affordable option for microbial lipid production due to abundant nitrogen sources in raw materials. The phosphorus limitation significantly impacts cellular metabolism and physiology, as it plays a crucial role in biomolecules like DNA, RNA, ATP and phosphorylated proteins (Wang et al. 2018). From the proteomics analysis of *Rhodosporidium toruloides* under phosphate-depleted and phosphate-rich conditions, the ribosome production and TCA cycle were downregulated in response to phosphate limitation, as well as the metabolites like AMP became dephosphorylated under phosphate stress, and NADPH was limited due to reduced pentose phosphate pathway and transhydrogenase cycle fluxes (Wang et al. 2018).

Besides increasing total lipid content, the growth temperature can affect the composition and saturation level of fatty acids in the depositing TAG (Ratledge 2004). The current findings indicated that when the temperature was lowered to 7 °C, linoleic acid (LA) was significantly increased in both yeast isolates, whereas palmitic acid was significantly decreased. These findings are in agreement with previous studies on *Rhodotorula* spp. (Granger et al. 1993; Amaretti et al. 2010). The drop in temperature to 5 °C also enhanced linoleic accumulation in *R. glutinis* (Granger et al. 1993). Generally, lowering the temperature below the optimal growth temperature increases the lipid content and thus affects lipid composition. Unsaturated FAs have a lower melting point than saturated FAs, and short-chain FAs have a lower melting point than long-chain FAs. That's why the ratio of linoleic acid to oleic acid (LA: OA) rises when the temperature drops (Suutari and Laakso 1994).

In agreement with these earlier observations, compared to 7 °C and 15 °C, the current results demonstrate a significant increase in carbon chain elongation at 26 °C. The short lipid profiles terminate at low temperatures with C18-carbon fatty acids, especially oleic and linoleic acids. In contrast, extended lipid profiles consisting of C20 and C22 FAs were detected at 26 °C in both HGM and HGM + VIT media (Table 2). This trend was previously observed when *L. elongisporus* was incubated at 26 °C on Basal Defatted Medium (BDM) (3% glucose), significant levels of 15-Docosenoic acid (C22:1, ω 7) and Tricosanoic acid (C23:0) were found as 12.12% and 21.49%, respectively, in its FAs profile (Adel et al. 2021). This trend confirmed the positive influence of high temperatures on the elongation mechanism in PUFA production in *L. elongisporus*.

According to the data shown in Table 2, the predominant FAs in yeast isolates were oleic (18:1, ω9), palmitic (16:0), stearic (18:0), and linoleic (18:2, ω 6). α-linolenic (18:3, ω 3), lignoceric (24:0), palmitic (16:1, ω 7), behenic (22:0), myristic (14:0), and arachidic acids (20:0) were among the minor FAs. As previously discovered in certain strains of *Saccharomyces* spp., *Rhodosporidium* spp., and *Rhodotorula* spp., these predominant FAs in common yeast strains, but other FAs were identified in trace amounts (Amaretti et al. 2010; Fakankun et al. 2019; Maza et al. 2020 and 2021). Some PUFAs were unexpectedly detected at 26 °C, including C18:3 (ω 3, ALA), C18:4 (ω 3, SDA), C20:5 (ω 3, EPA), C16:4 (ω 1 6, 9, 12, 15-hexadeca tetraenoic acid), C16:3 (ω 4, Hexadeca trienoic acid), and C16:4 (ω 3, Hexadeca tetraenoic acid). C18:4 (ω 3, SDA) was also suggested to be abundant in the lipid profiles of marine microalgae and terrestrial plants (Guil-Guerrero 2007). C18:3 (ω 3, ALA) has also been reported to be produced by marine *Rhodotorula* sp (Gupta et al. 2012). On the other hand, C16:4 (ω 3, Hexadeca tetraenoic acid), the principal precursor FA in the Docosahexaenoic Acid (DHA) denovo pathway of marine green algae, and C16:3 (ω 4, Hexadeca trienoic acid), which is present primarily in the FA composition of Menhaden oil, were observed in the lipid composition of *L. elongisporus*. Moreover, Table 2 shows that both yeast strains contained C16:4 (ω1, Hexadeca tetraenoic acid) in concentrations ranging from 0.44 to 3.83%, which is sufficient evidence to confirm the marine habitat of the isolates since marine oil is the sole source of these fatty acids (Dugo et al. 2012). In addition, C20:1 (ω 11, Gadoleic acid; cis-9-Eicosenoic acid) has been observed in Atlantic salmon FAs content (Routray et al. 2018).

Long-chain polyunsaturated fatty acids (LC-PUFAs), such as Eicosapentaenoic acid (EPA) and Eicosadienoic acid (EDA), were found in *L. elongisporus* and *R. mucilaginosa* at previously unreported levels of 19% and 19.6%, respectively. From a nutritional standpoint, human colostrum showed higher amounts of C20:2 (ω-6, EDA) 1.17% as compared to transitional 0.73% and mature milk 0.6% but C20:5 (ω-3, EPA) concentrations showed an opposing pattern from colostrum 0.51% to mature milk 0.87% (Guo 2020).

When cultivated in suitable culture conditions, microorganisms such as marine algae, certain fungi, and bacteria naturally produce EPA, which has recognized therapeutic and nutritional benefits (Shah et al. 2022). Conversely, until now, only the genetically modified yeast was able to synthesize LC-PUFAs (ω-3 and ω-6 FAs), which were created by introducing and expressing heterologous genes that encode the ω-3/ω-6 biosynthesis pathway in the oleaginous host (Gemperlein et al. 2019; Jovanovic et al. 2021). In 2006, DuPont (Wilmington, USA) achieved the commercial production of lipids from yeast through genetic modification. The genetically modified (GM) yeast strain accumulated 35% total lipid content, with 15% being EPA. Subsequently, the lipids were manufactured by CPKelco and marketed in the USA as NewHarvest™ EPA oil for human consumption and yeast biomass as animal feed under the Verlasso® brand in partnership with AquaChile (Puerto Montt, Chile) to produce salmon enriched with EPA. However, the process faced consumer criticism due to hexane extraction solvents and genetic modification technologies, potentially limiting market success and social acceptance (Abeln and Chuck 2021).

EPA can be produced by a mutant strain of *Yarrowia lipolytica* at 15% of DCW (MacKenzie et al. 2010; Xue et al. 2013a and Xue et al. 2013b; Yuan and Alper 2019). Hence, *L. elongisporus* is regarded as the first marine wild yeast strain to generate a considerable quantity of EPA at 10% of DCW. Furthermore, because EDA is a crucial intermediary FA in the Δ^−9^ elongase/Δ^−8^ desaturase pathway of EPA formation, *R. mucilaginosa* may be a promising future candidate yeast for EPA or DHA production. Even though there are a lot of concerns about using *L. elongisporus* and *R. mucilaginosa *in the food and pharmaceutical industries due to their potency to cause infectious diseases, related studies confirmed that both strains emerge as opportunistic human pathogens that cause nosocomial infections in immunocompromised patients (Tsui et al. 2008; Butler et al. 2009; Badr et 2021). According to our confirmation results from the blood agar hemolysis test and micromorphology test, gamma hemolytic phenotype was shown by both yeast strains, as well as the absence of hyphal growth form, which was the most invasive form in virulence mechanisms (ElMekawy et al. 2013; Mayer et al. 2013).

According to current findings, the two-stage batch fermentation method seems to boost both the total biomass and total PUFA content of yeast isolates (34.33 and 32.46%) (Tables 2 and 3). In this situation, it is possible that during the second stage of fermentation, old yeast cells save all energy for respiration and catabolize the carbon source to produce ATP molecules and lipid accumulation. The extra energy (accumulated ATP) is transformed into high-energy FA molecules that may be stored as TAG and natural lipids. The yeast cell seems to further modify its lipid composition by increasing the degree of unsaturation and elongation as a response to incidental stress brought on by the low substrates and the start of starvation after a long time of incubation (28 days) under excellent aeration conditions towards storage energy-dense PUFAs biosynthesis. Our findings support entirely the prior findings that showed that when OY transitions to low-carbon resources, they produce biomass at the expense of the accumulated fat (like the beginning of the first stage when glucose is 20 g/l). An increase in lipid synthesis causes an expansion of glucose catabolism through the pentose-phosphate pathway when nitrogen and carbon are limited. Furthermore, the dehydrogenation of lipids may be enhanced in the absence of a carbon resource (like the end of second stage fermentation) (El Baz et al. 2011; Ageitos et al. 2011).

Based on the current study, there was an inverse relationship between FA unsaturation and elongation and the level of glucose in the culture medium, as evidenced by the sharp decline of glucose content with both yeast isolates from 8 to < 0.2% at the end of the two-stage batch culture. This finding is consistent with Amaretti et al. (2010), who reported that the total PUFAs of *R. glacialis* DBVPG 4785 was increased by decreasing the glucose concentration at different temperatures (Amaretti et al. 2010). In addition, Deryabina et al. (2022) supposed that one of the factors providing great adaptability upon substrate depletion (0.2% glucose) is the shift of synthesis towards PUFAs after increasing unsaturated FAs in membrane phospholipids during the adaptation of *Endomyces magnusii* yeast in long-lasting cultivation and substrate restriction (Deryabina et al. 2022).

The area of bioprocessing that has received the least attention is downstream processing for oil extraction. Despite this, no standard technique can maximize oil recovery for all microbes. Consider the following observations while working with yeast lipids: (1) The yeast cell wall is mainly resistant to solvents; (2) Yeast lipids are membrane-bound or protected by phosphatides and proteins; (3) Yeast lipids are polar and nonpolar, requiring both solvent types for extraction. Therefore, we developed a simple cell disruption technique to improve total free FAs extraction from the tested marine isolates, in which osmotic pressure and pressure extrusion were used to efficiently break down the rigid cell wall, followed by solvent extraction. Such methods may reduce the risk of overheating induced by other mechanical processes like sonication, bead milling, and grinding, which may accelerate PUFA oxidation and limit PUFA extraction. Consequently, we extracted significant amounts of EPA and EDA from both marine yeasts (19% and 19.6%, respectively).

In addition, using a two-stage culture fermentation method boosted lipid accumulation, as reported frequently (Lorenz et al. 2017). In the second stage, the dramatic rise in lipid accumulation in yeast cells might result from the energy savings necessary for reproduction and biomass. It is decreasing the toxic waste produced compared to the first growth phase, which triggers cell death. In addition, the avoidance of budding or fission reduces the presence of scars, reducing the degree of cell wall rigidity and improving the efficiency of lipids extraction. Consequently, the total PUFA extraction for both yeast isolates rose significantly and reached its maximum (32.46 and 34.33%) at 26 °C (Table 2). Even though a two-stage culture technique allows lipid accumulation to be separated from the growth phase, increasing the total biomass of yeast isolates and the total PUFA content, the long incubation period still presents a challenge for this kind of fermentation. That might be because our psychrophilic yeast strains, despite their high lipid yield, grow slowly at 26 °C.

However, fish oil is easily oxidized and often associated with an unpleasant taste and odor. Moreover, the availability of ω-3 fatty acids depends on factors like season, harvest location, fish species, and its primary food source, mainly marine algae and protists. Overfishing and global warming contribute to marine biodegradation; in response to high temperatures, microalgae produce less ω-3 desaturated fatty acids and more saturated fatty acids. Vegetable oils like corn, soybean, and palm oil offer alternative sources for PUFAs, but they only synthesize Cl8 PUFAs like stearidonic acid (SDA, 18:4ω3) and α-linolenic acid (ALA, 18:3ω3) due to a lack of essential enzymes. The microbial production of PUFAs has gained increasing interest as a sustainable alternative to plant and animal sources. Microbes offer distinct advantages over traditional sources, including diverse substrate utilization and independence from climatic conditions. Their rapid growth and simplistic nature render microorganisms’ applicable models for elucidating PUFA biosynthetic pathways and optimizing production. Oleaginous microorganisms, accumulating over 20% of lipids, synthesize and store considerable fatty acid reserves, including PUFAs. Lipid profiles typically feature C4-C28-saturated and unsaturated fatty acids. Based on composition, oleaginous microbes show promise for biodiesel and nutraceutical applications. Certain yeast species have been shown to accumulate significant cellular lipid reserves, making them promising platforms for manufacturing biofuels and oleochemicals like *Yarrowia*, *Rhodotorula*, and *Lipomyces* with lipid weight fractions exceeding 25% of their biomass. Their rapid growth rates, doubling times often under an hour, allow faster productivity compared to slower-growing plants, algae, or fungi. Additionally, yeast cultivation is less influenced by environmental variables and more amenable to large-scale processing. Oleaginous yeasts can accumulate up to 65% of their weight as oil and reach high densities over a week, outpacing algae yields 100-fold. Common yeasts studied include *Yarrowia lipolytica*, *Lipomyces starkeyi*, *Rhodotorula toruloides*, *Cutaneotrichosporon oleaginosus*, and *Rhodotorula. glutinis* (Santamauro et al. 2014; Abeln and Chuck 2021). Further engineering of cultivation conditions and process development would be needed to enable economically viable scale-up while addressing contamination and equipment corrosion concerns.

In conclusion, overall results demonstrate a significant production of lipids, particularly PUFA, by both wild yeast strains in the absence of nitrogen source at different incubation temperatures. Using a two-stage batch culture system does exhibit some advantages in enhancing the intracellular LC-PUFA production by both yeast isolates over standard batch systems. This is the first time to report the production of LC-PUFAs such as EPA and EDA in significant amounts of 19 and 19.6% by the wild Ascomycetes yeast (*Loddoromyces elongisporus*) and the Basidiomycetes yeast (*Rhodotorula mucilaginosa*), respectively at 26 °C. These findings indicated the success of the current strategy to induce the elongation and desaturation mechanisms by both strains. Given public reservations regarding genetically engineered organisms, the explored indigenous oleaginous yeast isolates have innate lipid biosynthesis capacities without recombinant DNA modification. This addressing concerns over transgenic inputs in food and consumer products. Their efficient utilization of simple carbon sources and accumulation of nutritionally/therapeutically relevant lipids are technologically and commercially appealing and serve as attractive alternative production platforms for long-chain PUFAs. The current study provides the first step towards exploiting the biotechnological potential of marine yeasts for long-chain PUFA production. Continued study of additional medium components, alternative nutrient limitation strategies, and statistical experimental designs holds promise to improve productivity and customize lipid profiles significantly. Achieving this could position marine yeast as a sustainable source of high-value oils.

### Future work

While the current approach effectively induced lipogenesis and produced significant amounts of EPA and EDA, there remain lots of challenges in reducing the cost of production, shortening the incubation time, and optimizing to improve yield, composition, and productivity achieving through the design of experiment techniques response surface methodology. Hence, critical parameters affecting yeast lipid synthesis need to be evaluated and investigated in future work.

## Data Availability

All datasets generated for this study are included in the article.

## Abbreviations

3T3-L1: Adipocytes 3T3-L1 cell line, derived from 3T3 cells
ALA: α-Linolenic acid
ANOVA: Analysis of variance (statistical test)
ATP: Adenosine triphosphate
TCA: Tricarboxylic acid
BDM: Basal defatted medium
BLAST: Basic local alignment search tool
C: N: Carbon nitrogen ratio
CTAB: Cetyltrimethylammonium bromide
DCW: Dry cell weight
DHA: Docosahexaenoic acid
EDA: Eicosadienoic acid
EPA: Eicosapentaenoic acid
FAMEs: Fatty acid methyl esters
FAs: Fatty acids
FS: First stage growth medium
GLC or GC: Gas liquid chromatography
GM: Genetically modified
HGM: High glucose medium
HGM + Vit: High glucose medium with vitamins
HG-BDM: High-glucose basal defatted medium
ITS: Internal transcribed spacer
LA: Linoleic acid
LA: OA: Linoleic acid to oleic acid
LCFAs: Long chain fatty acids
LC-PUFAs: Long-chain polyunsaturated fatty acids
MALDI-TOF: Matrix-assisted laser desorption ionization–time of flight mass spectrometry
OY: Oleaginous yeast
OA: Orotic acid
PCR: Polymerase chain reaction
PUFAs: Polyunsaturated fatty acids
SCO: Single-cell oils
SDA: Stearidonic acid
SFAs: Saturated fatty acids
SPSS: Statistical Package for the Social Sciences software
SPV: Sulfo phospho vanillin
SS: Second stage fermentation medium
TAGs: Triacylglycerides
TFA: Total fatty acids
UNSFAs: Unsaturated fatty acids
Vitamin B1: Thiamine
Vitamin B12: Cyanocobalamine
Vitamin B3: Nicotinamide
Vitamin B5: Pantothenic acid
Vitamin B6: Pyridoxine hydrochloride
Vitamin B9: Folate
YPD: Yeast extract peptone dextrose

## Declarations

**Ethics approval and consent to participate**

This article doesn’t contain any studies with human participants or animals.
